# Chicken-Derived Pattern Recognition Receptor chLGP2 Inhibits the Replication and Proliferation of Infectious Bronchitis Virus

**DOI:** 10.3389/fmicb.2021.810215

**Published:** 2022-01-25

**Authors:** Kailu Wang, Pengfei Cui, Ruiqi Ni, Huiling Gong, Hao Li, Wenjun Yan, Xue Fu, Liang Chen, Changwei Lei, Hongning Wang, Xin Yang

**Affiliations:** ^1^Key Laboratory of Bio-Resources and Eco-Environment, Ministry of Education, College of Life Science, Sichuan University, Chengdu, China; ^2^Animal Disease Prevention and Food Safety Key Laboratory of Sichuan Province, Chengdu, China; ^3^Deyang Animal Disease Control Center, Deyang, China

**Keywords:** IBV, chLGP2, chTRBP, IFN-β, innate immunity

## Abstract

The widespread nature and economic importance of Infectious bronchitis virus (IBV) and interactions between IBV and the host immune response remain poorly understood. Understanding the mechanism of virus recognition via innate immunity can help resist IBV invasion. Retinoic acid-induced gene I-like receptor (RLRs) recognize virus RNA in virus infection, and LGP2 is a member of RLRs. According to the current studies, LGP2 exhibited certain inhibition in the virus, and there is a lack of investigation for chicken’s LGP2. It is important to figure out the role of chLGP2 in host immune recognition of IBV. Our results showed that chLGP2 inhibited the proliferation of IBV Beaudette in cells. Also, chLGP2 can identify and combine with IBV RNA. The domains of chLGP2 were separately expressed and inspired by related literature, and the chLGP2 K30A mutant was constructed. Our results suggested its structural integrity and the adenosine triphosphatase (ATPase) activity are critical for IBV inhibiting activity. chTRBP was selected after CO-IP and Mass spectrometry test. We found chTRBP and chLGP2 are the interacting partners and promote mutual expression. Our study showed that chTRBP could also suppress IBV infections via chLGP2, which provided a basis for future innate immunity research for IBV.

## Introduction

Infectious bronchitis virus (IBV) causing infectious bronchitis (IB) is a highly contagious disease in poultry. Infectious bronchitis is more common in chicks causing bronchial rales, sneezing, and coughing, while some IBV serotypes also lead to kidney or fallopian tube diseases. Infectious bronchitis virus infection significantly deteriorates the quality and quantity of the poultry industry causing huge economic losses every year. IBV is a single positive-stranded non-segmented enveloped RNA virus that belongs to the family of Coronaviruses and is capable of infecting various mammals and birds. Based on serotype and genome, coronaviruses can be divided into four subgenres, namely alpha, beta, gamma, and delta coronavirus (Cavanagh, 2007). Infectious bronchitis virus is classified as a γ-coronavirus that has ∼27.6 kb genome containing ten ORFs (Open Reading Frames): 5′-UTR-1a-1b-S-3a-3b-3c(E)-M-5a-5b-N-UTR-3′. The unstructured gene occupies about 2/3 at 5′UTR, while the structured gene occupies the rest at 3′UTR. The genome encodes four major structural proteins spike (S), membrane (M), nucleocapsid (N), and small membrane (E) (Brierley et al., 1987).

Cytoplasmic RLRs are the key recognition receptors for viral infections. retinoic acid-induced gene I-like receptor family (RLRs) recognize the RNA viruses and then mediate the production of type 1 IFN through signal transduction (Yoneyama and Fujita, 2009). LGP2, RIG-I, and MDA5 have different characteristics of RNA recognition and binding for different RNA viruses depending on structural features. For different types of RNA virus infections, RLRs play different recognition roles (Pippig et al., 2009). All three of RIG-I, MDA5, and LGP2 have a DexD/H box helicase domain and a C-terminal regulatory domain. In addition, RIG-I and MDA5 have a cysteine inflammatory protease recruitment activation domain (CARD) connected in series at the N-terminus, which interacts with the downstream adaptor protein MAVS to mediate signal transduction (Streicher and Jouvenet, 2019). Many studies have been conducted on RIG-1 and MDA5, but the role of LGP2 remains controversial.

LGP2 (Laboratory of genetics and physiology 2) is a key part of the RLRs that shares high homology with MDA5 and RIG-I (Rehwinkel and Gack, 2020). In lack of CARD, LGP2 cannot transmit signals downstream; however, a variety of virus infections can cause changes in the expression of LGP2. Also, some reports suggest that LGP2 exerts immunoregulatory effects on RIG-1 and MDA5 mediated antiviral signal transduction (Gack et al., 2007; Vitour and Meurs, 2007; Liu et al., 2013; Rodriguez et al., 2014; Parisien et al., 2018; Quicke et al., 2019). Several studies showed that LGP2 could be expressed in mammals, poultry, fish, and invertebrates, although the LGP2 gene sequence showed differences in different species; the gene size in the LGP2 open reading frame and the number of amino acids produced by translation remained similar, forming a DExD/H helicase domain and a C-terminal regulatory domain (C-terminal regulate domain, CTD). LGP2 helicase domain has adenosine triphosphatase (ATPase) activity, and the energy produced by ATP hydrolysis determines the LGP2 and RNA dissociation rate regulating LGP2 function (Bruns et al., 2014). The cryo-electron microscopy structure of chicken LGP2 (chLGP2) protein showed that it binds dsRNA through C-terminal regulate domain (CTD) to promote the filamentation of MDA5, producing a fast antiviral immune response (Bruns et al., 2014; Uchikawa et al., 2016). LGP2, as an RNA sensor in the cytoplasm, recognizes pathogenic RNA and plays an immunomodulatory role in various RNA virus infections. During viral infection, LGP2 promotes timely antiviral signals, inhibits viral infection, and suppresses antiviral immune responses, avoiding excessive immune response. In porcine, LGP2 promotes the binding of RIG-I/MDA5 and RNA, and its ATPase sites are not participating in this progress (Li et al., 2021). For the Sendai virus, antiviral signals are restrained by LGP2 in a manner associated with the C-terminus of TRAF and affect its ubiquitin ligase activity (Parisien et al., 2018). In addition, mammalian Dicer can cut virus-derived double-stranded RNA (dsRNA) efficiently *in vivo*, and the LGP2 regulates this process *in vivo* (Zhang et al., 2021). Thus it maintains antiviral immune homeostasis through various immune-regulatory methods. Moreover, in the lack of RIG-I in chicken cells (Zou et al., 2009; Barber et al., 2010), chLGP2 may have additional unknown roles under positive evolutionary selection.

TRBP helps RNA silencing under siRNAs/miRNAs affect. TRBP is a double-stranded RNA binding protein family member, including three dsRNA binding domains. The first two are responsible for RNA binding and the C-terminal dsRNA binding domain for combining Dicer, PKR (protein kinase R), PACT (PKR associated activator). TRBP (Trans-activation response RNA-binding protein), Dicer, and Ago2 together constitute RISC (RNA-induced silencing complex) (Chendrimada et al., 2005; Haase et al., 2005; Wang et al., 2012). Studies have shown that TRBP can also respond to oxidative stress by ERK and JNK mediated phosphorylation, and phosphorylated TRBP helps suppress PKR’s activity to avoid cell apoptosis (Chukwurah and Patel, 2018). Overexpression of TRBP promotes HIV-1 promoter and viral production, mainly caused by weakening the interaction between TRBP and PKR (Daniels and Gatignol, 2012).

Therefore, this study used an IBV model to analyze chLGP2 immunomodulatory roles in IBV infection, and we found that chLGP2 could inhibit IBV. Moreover, we found that chTRBP could interact with chLGP2. Thus chTRBP may participate in inhibiting IBV. Our results have certain reference significance for the future innate immunity research and prevention and control of IBV.

## Results

### chLGP2 Inhibited the Proliferation of Infectious Bronchitis Virus Beaudette Strain

**FIGURE 1:**
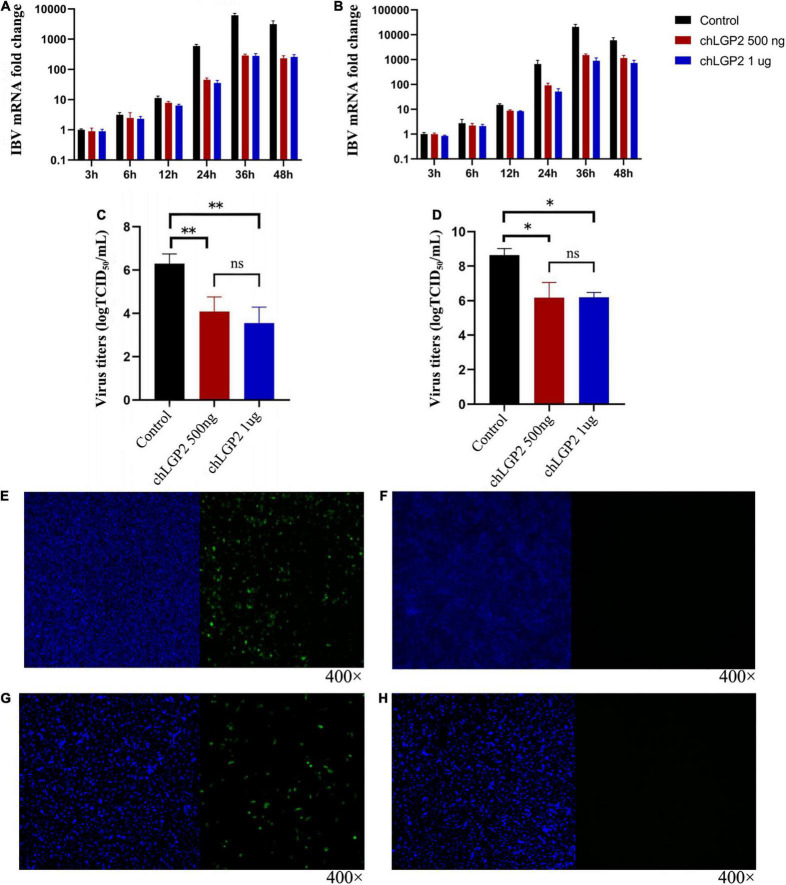
Expression of chLGP2 in HD11 and DF-1 cells and effect on IBV Beaudette strain. chLGP2 overexpression significantly inhibited IBV Beaudette replication in **(A)** HD11 and **(B)** DF-1 cells. Virus titers (TCID_50_) at 36 h post-infection in cell supernatant of **(C)** HD11 and **(D)** DF-1 cells. Expressions of **(E)** chLGP2 or **(F)** empty plasmid in HD11 cells. Expression of **(G)** chLGP2 and **(H)** empty plasmid in DF-1 cells. Blue fluorescence represents the nucleus, and green fluorescence represents the transfected chLGP2. Data are the mean values from three independent experiments, and error bars denote mean ± SDM. *P < 0.05; ^**^ *P* < 0.01; ns means not significant.

To figure out whether chLGP2 plays a role in inhibition, as shown in Figures 1E–H, compared to the control, chLGP2 was successfully expressed both in HD11 and DF-1 cells. The results showed that the overexpression of chLGP2 significantly inhibited the IBV replication in both DF-1 and HD11 cells (Figures 1A,B). The virus titer was calculated based on the TCID_50_ test; the cells and the cell supernatant were collected at 3 h, 6 h, 12 h, 24 h, 36 h, 48 h post-infection. In both cell lines, the virus titer of the control group was significantly higher than that of the chLGP2 overexpression (Figures 1C,D). In addition, the inhibitory effect of chLGP2 on the IBV Beaudette strain was weaker in DF-1 cells than in HD11 cells.

### chLGP2 Showed Differences in Distinct Cell Lines and Virus

**FIGURE 2:**
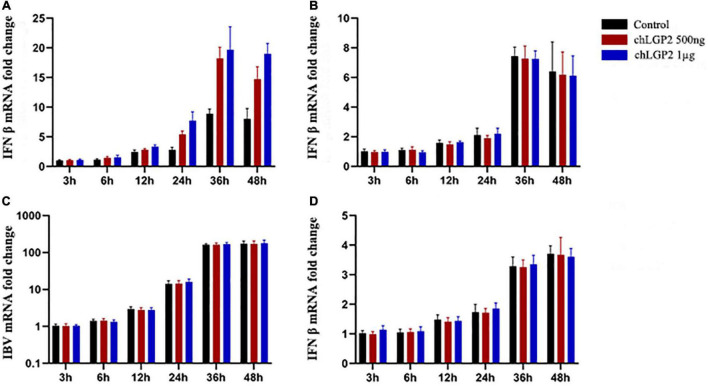
The cell and virus-specific effect of chLGP2. Post-IBV Beaudette infection, chLGP2 overexpression significantly upregulated IFN-β expression in **(A)** HD11 cells but not in **(B)** DF-1 cells. **(C)** chLGP2 overexpression did not inhibit the replication of IBV M41 in DF-1 cells. **(D)** chLGP2 did not affect the IFN-β expression during IBV Beaudette infection in DF-1 cells. Data are the mean values of three independent experiments, and error bars denote mean ± SDM.

The cells were collected at different time points and subjected to qRT-PCR to detect the cellular expression level of IFN-β. After IBV Beaudette infection, chLGP2 significantly promoted the expression of IFN-β in HD11 cells but not in DF-1 cells (Figures 2A,B). This result suggests that in addition to IFN-β up-regulation, chLGP2 can also inhibit the IBV replication in some other ways, as in DF-1 cells, indicating a cell-specific role of chLGP2.

Compared with the IBV Beaudette strain, the IBV M41 is a more virulent strain. In addition to chicken embryonic fibroblasts, chicken embryo kidney cells, and other chicken primary cells, IBV M41 can temporarily replicate in DF-1 cells. Total cell RNA extraction and qRT-PCR detection at the indicated 6-time points showed that the IBV M41 mRNA replication was low, unlike the IBV Beaudette strain, which replicated exponentially in DF-1 cells. Notably, there were no significant differences in the amount of IBV M41 replication between the chLGP2 overexpression and the control groups (*P* > 0.05). Also, after IBV M41 infection, chLGP2 had no significant effect on IFN-β expression in DF-1 cells (*P* > 0.05), suggesting that chLGP2 maybe not be involved in the inhibition of IBV M41 strain in the DF-1 cells (Figures 2C,D).

### ATPase Activity of chLGP2 Is Important for Infectious Bronchitis Virus Inhibition

**FIGURE 3:**
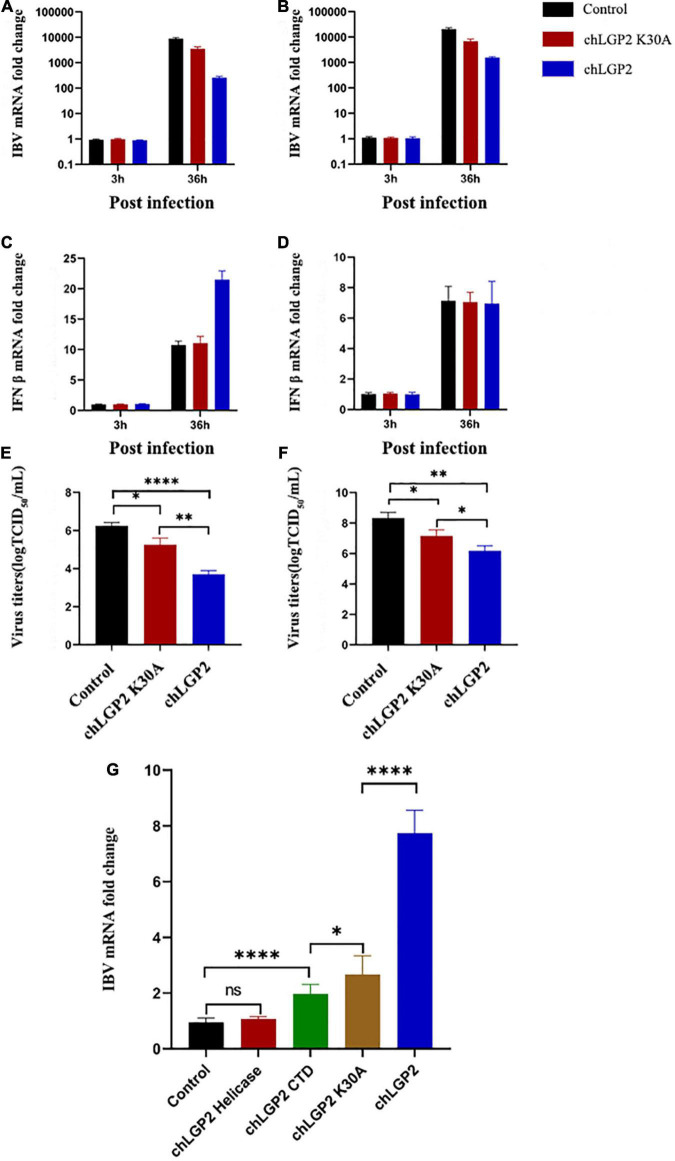
Effect of chLGP2 ATPase activity and individual domains on IBV RNA binding. Compared with the control, chLGP2 K30A inhibited the replication of IBV in both **(A)** HD11 and **(B)** DF-1 cells at 36 h post-infection. However, chLGP2 K30A did not affect the production of IFN-β in **(C)** HD11 and **(D)** DF-1 cells. The mutant decreased the titer of the IBV B strain, but the inhibitory effect was weaker than that of wild type in both **(E)** HD11 and **(F)** DF-1 cells. **(G)** The IBV RNA binding ability of different chLGP2 constructs was as follows: chLGP2 > K30A > CTD > Helicase. Data are the mean values of three independent experiments, and error bars denote mean ± SDM. * *P* < 0.05; ^**^ *P* < 0.01; ^****^ *P* < 0.0001; ns means not significant.

**FIGURE 4:**
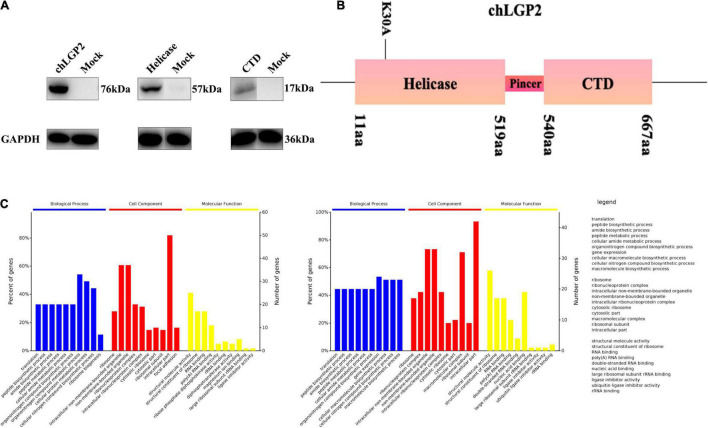
The interaction between chLGP2 and chTRBP and the expression of chLGP2 domains. **(A)** Extracellular expression of chLGP2, Helicase domain, and CTD. **(B)** Schematic of different domains and the mutant K30A of LGP2. **(C)** GO enrichment analysis of chLGP2 bound proteins based on mass spectrometry results.

In this part, the IBV RNA binding of chLGP2, chLGP2 helicase, chLGP2 CTD, and chLGP2 K30A proteins was investigated. Figure 4B shows the schematic diagram of each domain of chLGP2 and the K30A mutant of chLGP2. The results of RNA pull-down and qRT-PCR revealed that both helicase and CTD domains could bind to IBV RNA. Importantly, chLGP2 showed the highest binding, while the helicase domain showed a weaker binding (Figure 3G). chLGP2 acts as a foreign RNA receptor in chicken cells, in which CTD mainly binds to IBV RNA. Meanwhile, the helicase domain and CTD work cooperatively, while ATPase activity helps in viral RNA recognition.

We overexpressed each domain to explore the role of chLGP2 domains in IBV inhibitory function and verify the corresponding expression by Western blotting (WB) (Figure 4A).

**FIGURE 5:**
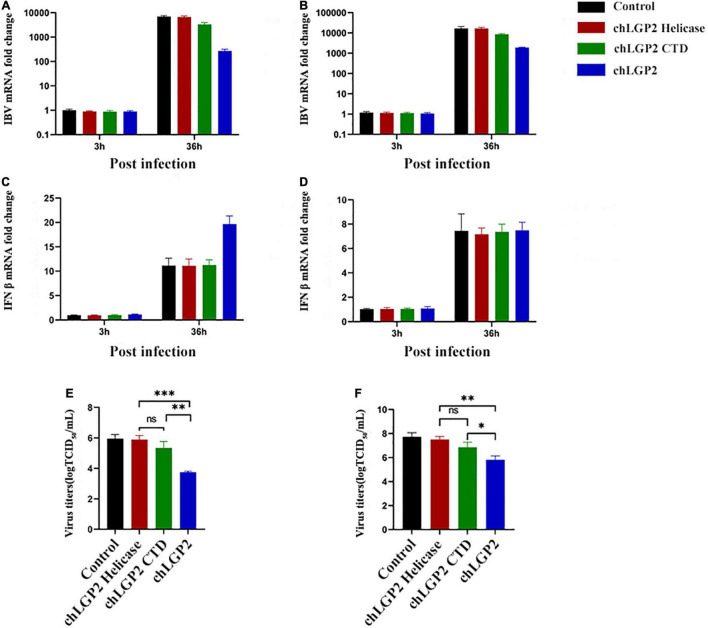
The IBV inhibitory roles of chLGP2 domains in different cells. Compared with the helicase domain, the CTD domain of chLGP2 showed a certain inhibitory effect on IBV both in **(A)** HD11 and **(B)** DF-1 cells. **(C)** In HD11 cells, the individual domains of chLGP2 did not change the level of IFN-β, while in **(D)** DF-1 cells, the domains and the full chLGP2 showed almost the same effect. The CTD domain of chLGP2 inhibited the virus titer of IBV to a certain extent, both in **(E)** HD11 and **(F)** DF-1 cells. Data are the mean values of three independent experiments, and error bars denote mean ± SDM. * *P* < 0.05; ^**^ *P* < 0.01; ^***^ *P* < 0.001; ns means not significant.

Results showed that overexpression of the chLGP2 helicase domain did not significantly reduce the replication of the IBV Beaudette strain, while the over-expression of chLGP2 CTD showed a certain inhibitory effect (Figures 5A,B). Meanwhile, the virus replication was still significantly higher compared to that of full chLGP2 protein. Moreover, the individual domains of chLGP2 did not significantly affect the expression of IFN-β (Figures 5C,D). The cell supernatants collected at 36 h post-transfection also showed that chLGP2 helicase and CTD overexpression did not significantly reduce the IBV titer (Figures 5E,F). So, the two domains must work jointly to function.

The ATPase activity of LGP2 plays an important role in its function involving K30 residue. Accordingly, the 88^th^ and 89^th^ bases of the chLGP2 open reading frame were double mutated to GCA (K) and AAA (A) to generate chLGP2 but lacked ATPase activity.

The qRT-PCR results showed that overexpression of chLGP2 K30A inhibited the replication of IBV. However, the effect was significantly lower than wild-type (Figures 3A,B,E,F). Furthermore, chLGP2 K30A did not promote the production of IFN-β as reflected by Figures 3C,D. For IBV inhibition, chLGP2 ATPase activity is essential for upregulating the expression of IFN-β, especially in HD11 cells. However, the loss of ATPase activity in chLGP2 K30A still inhibited IBV replication, which suggests that chLGP2 can inhibit IBV proliferation in multiple ways.

### chLGP2 Could Interact With chTRBP

Based on the results above, we believe that chLGP2 functions differently and might involve other factors. The DF-1 and HD11 cells were plated on 6-well cell plates and transfected with chLGP2 plasmid (1 μg/well). After 24 h of IBV Beaudette strain infection, the cell lysates were collected and added with 40 μL anti-Flag affinity gel magnetic beads to analyze protein mass spectrometry (MS).

The MS results showed that no IBV protein was bound to chLGP2. A total of sixty-five proteins bind to chLGP2, seventeen of which are shared in both cells, like calreticulin, TRBP, endoplasmic reticulum chaperone protein Bip (Supplementary Figures 1, 2), and studies have shown they exert in virus infection to a certain extent. Furthermore, Gene Ontology (GO) analysis results showed that differences existed in protein composition and molecular function in HD11 and DF-1 cells (Figure 4C). A previous study showed that TRBP could interact with LGP2 in virus infection, inhibiting miRNA maturation and promoting apoptosis. However, so far, the role of chTRBP in IBV virus infection is unknown. Accordingly, a chTRBP eukaryotic expression carrier marked with HA at the C-terminus was constructed (cDNA3.1-chTRBP-HA).

Flag-tagged chLGP2 plasmid and HA-tagged chTRBP plasmid were co-transfected into the HD11 cells to verify the specific binding between chLGP2 and chTRBP. The cells were collected 36 h after co-transfection. WB analysis revealed that chLGP2 and chTRBP showed specifical binding in HD11 cells (Supplementary Figure 3). So maybe the chTRBP is a crucial factor for chLGP2’s inhibition.

### chTRBP Showed an Inhibitory Effect on Infectious Bronchitis Virus Replication

**FIGURE 6:**
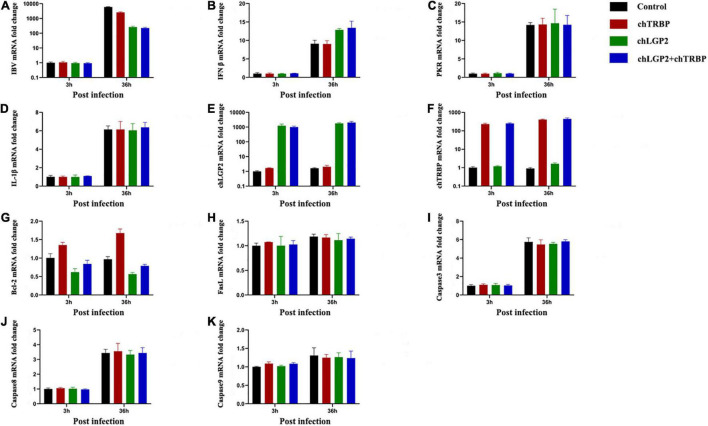
Effects of chTRBP overexpression and co-expression of chTRBP and chLGP2 on IBV replication and expression of immune-related genes: **(A)** IBV; **(B)** IFN-β; **(C)** PKR; **(D)** IL-1β; **(E)** chLGP2; **(F)** chTRBP; **(G)** Bcl-2; **(H)** FasL; **(I)** Caspase3; **(J)** Caspase8; **(K)** Caspase9. Data are the mean values from three independent experiments, and error bars denote mean ± SDM.

chTRBP, chLGP2, or chTRBP + chLGP2 were overexpressed in HD11 cells, and then the cells were infected with IBV to examine the relationship between chTRBP and IBV infection. The cells were collected at 3 h and 36 h post-infection. IBV replication and the expression of related anti-IBV immune genes were detected by qRT-PCR. As shown in Figure 6, overexpression of chTRBP inhibited IBV replication in HD11 cells, but chTRBP did not affect the expression of IFN-β and IL-1β. The same was observed for PKR. In the control group of cells infected with IBV, chTRBP upregulated the expression of chLGP2, vice versa, which suggests that chLGP2 and chTRBP promote expression of each other, which may be related to the inhibitory effect of chTRBP on IBV. In addition, chLGP2 inhibits the expression of apoptosis-related gene Bcl-2, but chTRBP upregulates Bcl-2. Also, there is no significant effect on the expression of FasL, caspase3, caspase8, and caspase9, suggesting interrelated roles of chTRBP and chLGP2 involving apoptosis in IBV-infected HD11 cells, which requires further exploration.

## Discussion

Infectious bronchitis virus (IBV) is an acute and highly infectious chicken upper respiratory disease that causes bronchitis (Boltz et al., 2004), leading to serious economic losses to the poultry industry worldwide (Bezuidenhout et al., 2011; Jackwood, 2012). The incidence of new recombinant and variant IBV strains is rising (Sjaak de Wit et al., 2011). A single serotype IBV vaccine lacks good cross-protection limiting vaccine efficiency for the control of IB (Jackwood et al., 2012). Understanding the replication and pathogenic mechanism of IBV can highlight new prevention and treatment methods against infectious bronchitis diseases in avians and be used as a reference for other coronavirus diseases. After IBV entry into the cell, the host cell’s immune recognition system develops antiviral immunity. The double-stranded RNAs (dsRNAs) and single-stranded RNAs (ssRNAs) produced by IBV replication are recognized by the pattern recognition receptors located in the cytoplasm involving melanoma differentiation-associated protein (MDA5) and the Toll-like receptors located in the endosome 3 (Tool like receptor 3, TLR3), TLR7 recognition, and then through signal transduction to produce interferon (Interferon, IFN), pro-inflammatory cytokines inhibit the replication of IBV (Kint et al., 2015; He et al., 2016). chLGP2 is a cytoplasmic protein that encodes a DExD/H helicase, which can play a variety of immunomodulatory effects during RNA virus infection. The study explored the role of chLGP2 protein in the process of IBV infection.

In this study, chLGP2 was overexpressed in HD11 and DF-1 cells, and then the cells were infected with IBV Beaudette or M41 strain. The results showed that chLGP2 significantly inhibited the proliferation of IBV Beaudette. However, in DF-1 cells, chLGP2 had no significant effect on the replication of the IBV M41 strain, which may be due to the low replication of the IBV M41 strain in DF-1 cells, which may not be enough to activate the antiviral effect of chLGP2. There could be a unique immune escape in the cell process, which antagonizes the function of chLGP2. Also, the production of intracellular IFN-β showed a significant difference, indicating a different antiviral mechanism of chLGP2.

The protein structure plays a decisive role in its function. chLGP2 is composed of a helicase domain and a CTD; the helicase domain has ATPase activity that binds to dsRNA. Studies showed that the combination of Paramyxovirus V protein and LGP2 inhibits the activation of MDA5 via LGP2, directly inhibiting the MDA5 mediated antiviral signal reducing the production of IFN and virus replication (Parisien et al., 2009; Rodriguez and Horvath, 2013, 2014). CTD functions as a link between pathogenic RNA and immune regulation. PARISIEN J P et al. showed that LGP2 specifically binds to Dicer through the C-terminal regulatory domain to inhibit RNA interference during viral infection (Parisien et al., 2018). Notably, the CTD of LGP2 and RIG-I has a high sequence homology, and the CTD of LGP2 can competitively recognize and bind to foreign RNA and inhibit RIG-I-mediated signal transduction (Saito et al., 2007). There have been many studies on LGP2 regulating antiviral immunity, but only a few studies investigated the role of its domains in isolation. Our study showed that these two domains alone failed to promote the production of IFN-β in HD11 cells. Moreover, the IBV inhibitory effect of expressing any one domain alone was not comparable to the full-length chLGP2 protein, suggesting cooperativity between the two domains of chLGP2.

ATP is a universal energy carrier in cellular activities and an important basis for many protein functions (Kenniston et al., 2003). Hei and Zhong (2017) showed that LGP2 could promote the production of IFN and inhibit virus replication during hepatitis C virus infection. They constructed an LGP2 K30A mutant to inactivate its ATPase activity and found that the mutant failed to induce IFN production (Hei and Zhong, 2017). Similarly, we obtained a chLGP2 K30A mutant and showed that the mutant does weaken the effect of chLGP2 but does not completely abolish the IBV inhibitory activity. Therefore, chLGP2 may also inhibit the virus in other ways and not entirely rely on its ATPase activity.

Several studies analyzed the proteins that can specifically bind to LGP2 in the process of viral infection to understand the mechanism of LGP2. LGP2 can interact with endonuclease DICER1, PKR, PACT, NF-κB inhibitor (NKPF), and 5′-3′endo- nuclease 2 (XRN2). The interaction between LGP2 and PACT plays an important role in regulating RLRs signaling (Sanchez David et al., 2019). Since chLGP2 showed distinct effects in HD11 and DF-1 cells, this study analyzed the protein components that interact with chLGP2 in HD11 and DF-1 cells. We found certain differences, such as chLGP2 can interact with heat shock proteins H1 and A8 in HD11 cells, inducing IFN production through non-classical/alternative pathways to inhibit viral replication (Li et al., 2012), which again confirms the complexity of the LGP2 function.

Among the many interacting protein partners, chTRBP was selected for further investigation. Tomoko Takahashi et al. found that the helicase domain of LGP2 specifically binds to the dsRNA binding domain of TRBP to inhibit its pre-miRNAs recruitment function, thereby inhibiting the maturation of miRNAs (Takahashi et al., 2018). Another study showed that the LGP2-TRBP combination could promote cell apoptosis during Sendai virus infection. miRNA sequencing technology revealed that the LGP2-TRBP combination inhibited the maturation of cell apoptosis inhibitor miR-106b (Takahashi et al., 2020). In other antiviral innate immune responses, TRBP plays a negative regulatory role inhibiting the activation of PKR and promoting the replication of HIV-1 (Sanghvi and Steel, 2011). In this research, chTRBP inhibited the replication of IBV in HD11 cells without affecting the expression of PKR, contrary to previous studies where TRBP played a negative regulatory role during viral infection, which can be attributed to the natural deletion of the PACT gene in chicken cells. As a result, chTRBP compensates for the function of PACT through positive selection, thereby exerting an antiviral effect. Overexpression of chTRBP did not upregulate the IFN and IL-1β genes, but chTRBP and chLGP2 mutually promoted the expression of each other, which may be a reason behind chTRBP mediated inhibition of IBV replication. Down-regulation of Bcl-2 promotes apoptosis through the mitochondrial pathway (Procko et al., 2014). In this study, chLGP2 exerted an inhibitory effect on Bcl-2, while chTRBP upregulates Bcl-2. Previously, Han Xiaoxiao and others demonstrated that IBV induces HD11 cell apoptosis by activating the Fas/FasL-mediated death receptor pathway and the Bcl-2 family mediated mitochondrial pathway (Han et al., 2017). Therefore, chTRBP and chLGP2 regulated apoptosis in IBV-infected HD11 cells require further exploration. In the future, we can screen for chLGP2 specific agonists and explore their anti-IBV effect in chickens as potential immune adjuvants.

## Materials and Methods

### Cells, Viruses, and Antibodies

Chicken macrophage cell line HD11 was presented by Professor Jiao Xin’an from Yangzhou University and maintained in Dulbecco’s modified Eagle’s medium (DMEM) (HyClone), supplemented with 10% fetal bovine serum (FBS) (Gibco) and 1% penicillin-streptomycin (DMEM growth medium). Chicken fibroblast cell line DF-1 was from our laboratory and maintained in DMEM (HyClone), supplemented with 10% FBS (Gibco) and 1% penicillin-streptomycin (DMEM growth medium).

Both IBV Beaudette strain and IBV M41 strain (standard strain) were from our laboratory. For WB and immunofluorescence assays, the Flag-tag mouse monoclonal antibody (Beyotime Biotechnology), HRP-labeled goat anti-mouse IgG(H + L) (Beyotime Biotechnology), GAPDH Mouse Monoclonal Antibody (Beyotime Biotechnology), and Alexa Fluor 350 labeled goat anti-rabbit IgG (H + L) (Beyotime Biotechnology) were used as primary and second antibodies, respectively.

### Plasmids Construction and Transfection

The C-terminus of the open reading frame sequence of the chLGP2 gene (NCBI accession number HQ845773) was fused with Flag tag (GATTACAAGGAT GACGACGATAAG) and cloned between the *Eco*RI and *Bam*HI restriction sites of the pcDNA3.1 vector. The open reading frame of chLGP2 is 2025 bp, encoding 674 amino acids. LGP2 comprises a helicase domain and a C-terminal regulatory domain connected by pincer. A Flag tag and a stop codon were added to the C-terminus of the helicase (1482 bp), and a start codon and a Flag tag were added to the N-terminus of the regulatory domain (612 bp) at the C-terminus. These were cloned using *Eco*RI and *Bam*HI in the pcDNA3.1 vector. The open reading frame sequence of the chTRBP gene (gene accession number: XM_015300349.2) was fused with a HA tag (TACCCATACGATGTTCCAGATTACGCT) at the C terminus and cloned using *Eco*RI and *Bam*HI. The above-designed cDNA sequences and plasmids were sent to Beijing Kinco Xinye Biotechnology Co., Ltd. for full gene synthesis. chLGP2 plasmids (500 ng/well and 1 μg/well) were transfected into HD11 cells and DF-1 cells using Lipofectamine™8000 Transfection Reagent (Beyotime Biotechnology); the empty plasmid (500 ng/well) was used as a negative control (Figure 1).

### RNA Extraction and Quantitative Real-Time PCR

Total RNA was extracted from cells using Trizol reagent (Invitrogen) and reverse-transcribed using a RevertAid First Strand cDNA Synthesis Kit (Thermo Scientific) following the manufacturer’s instructions. cDNA samples were subjected to polymerase chain reaction (PCR) amplification and electrophoresis to detect chLGP2 expression. The quantification of IFN-β and IBV N gene expression was quantified using SYBR green-based qRT-PCR on a CFX96 system (Roche, Basel, CH, Germany). Primers used were as follows: chicken IFN-β (Accession no.NM_001024836), F: GCTCTC ACCACCACCTTCTC and R: GCTTGCTTCT TGTCCTTGCT; IBV N (Accession no.AY851295), F: GAA GAAAACCAGTCCCAGA and R: TTACCAGCAAC CCA CA; chicken GAPDH (Accession no.NM_204305), F: CAT CACAGCCACA CAGAAG and R: GGTCAGGTCAACA ACAGAGA; chicken PKR (Accession no.NM_204487) F: CCTCTGCTGGCCTTACTGTCA and R: AAGAGAGGC AGA AGGAATAATTTGCC; chLGP2 (Accession no.HQ845773.1) F: AGCTCCACG GGTACCAACT and R: AGCTCCA CGGGTACCAACT; chTRBP (Accession no.XM_015300349.2) F: TGCAGGAGTATGGCACACGCAT and R: CGACGG TGACACGGAAGGTGAA; chicken BCL-2 (Accession no.NM_205339.2) F: TGT TTCTCAAACCAGACACCAA and R: CAGTAGGCACCTGTGAGATCG; chicken FasL (Accession no.AJ890143) F: GGCATTCAGTACCGTGACCA and R: CCG GAAGAGCACATTGGAGT; chicken IL-1β (Accession no.Y15006.1) F: CTG GGCATCAAGGGCTACAA and R: CGGTAGAAGATGAAGCGGGT; chicken Caspase3 (Accession no.NM_204725.1) F: TACCGGACTGTCATCTCGTTCAGG and R: ACTGCTTCGCTTGCTGTGATCTTC; chicken Caspase8 (Accession no.AY057939.2) F: GGAAGCAGTGCCAGAA CTCAGAAG and R: TTGTTGTG GTCCATGCACCGATAG; chicken Caspase9 (Accession no.BG711928.1) F: CCGAA G GAGCAAGCACGACAG and R: CATCTAGCATGTCAGC CAGGTCAC.

### Site-Directed Mutation

To obtain the chLGP2 K30A mutant, we designed amplification primers that can reverse pcDNA3.1-chLGP2-Flag in reverse PCR and introduced a mutation site in the primers (Forward primer: ACGGGCGCCGGCGCAAAACCCGCGCGGCTGTGCA, reverse primer: TTTTGCGCCGGCGCCCGTGCCGGCGCCC GTGGGCAG) synthesized by Beijing Kinco Xinye Biotechnology Co., Ltd. Mut Express MultiS Fast Mutagenesis Kit (Vazyme Biotech Co., Ltd) V2 was used to introduce continuous double base mutations in chLGP2 gene following the kit instructions. The sequence of the mutant plasmid was validated by Beijing Kinco New Industry Co. Ltd.

### Virus Titration

The cells were spread in a 96-well plate and cultured to 80% confluency. The cells were infected with IBV, and the cell supernatant was collected 36 h after infection, which was 10-fold serially diluted with Opti-DMEM, 8 replicate holes per gradient. Also. The cells with the same Opti-DMEM medium were set as a negative control. After 48 h, the cytopathic condition of each dilution was observed, and the number of lesions in each gradient was counted. The TCID_50_ value of IBV in the supernatant was estimated using the Reed-Muench method.

### SDS-PAGE and Western Blotting

The cells were harvested and lysed in RIPA lysis buffer (Thermo Scientific, contains protease inhibitors). An appropriate protein sample was mixed with the loading buffer and heated at 100°C for 15 min. The heat-denatured samples were resolved using pre-configured sodium dodecyl sulfonate-Poly- acrylamide gel electrophoresis (SDS-PAGE) gel at 120 V. The protein bands from the SDS-PAGE gel were transferred to the methanol-activated PVDF at 90 V for 1.5 h. After the transfer, the PVDF membrane was soaked in 5% skimmed milk in PBST (Phosphate Buffered Saline with Tween 20) at room temperature (RT) for 2–3 h or overnight. The blocking solution was removed, and the membrane was washed with PBST 3 times, 5 min each time. Then, the membrane was incubated with Flag-tag mouse monoclonal antibody (1:1000) and GAPDH mouse monoclonal antibody (1:1000) at RT for 2–3 h, followed by 3 times washing with TBST as earlier. Next, the membrane was incubated with HRP-labeled goat anti-mouse IgG (H + L) antibody (1:1000) at RT for 1–2 h. The membrane was washed 3 times with TBST for 5 min each, and ECL and DAB were used for color rendering. The image was developed using a ChemiDoc Touch Imaging System (Bio-rad).

### Indirect Immunofluorescence Experiment

An indirect immunofluorescence experiment was performed after cell culture for 24 h. The two 6-well cell culture plates with HD11 and DF-1 cells were removed from the cell incubator, and the cell supernatant was discarded. The plates were gently rinsed with PBS 3 times, 1 min each time. Next, 1 mL of 4% formaldehyde fixing solution was added to each well at 4°C for 15 min. Then, the fixative solution was removed, and the wells were washed with PBS 3 times, 1 min each time. 1 mL of immunostaining blocking solution was added to each well at 4°C. Subsequently, the immunostaining blocking solution was removed, and again 3 times washing was done with PBS. 1 mL of diluted Flag monoclonal antibody solution (500-fold dilution) was to each well for incubation at 37°C for 2 h. After rising with PBS, 1 mL of secondary antibody (diluted 500 times) was added to each well in the dark for 2 h incubation. After PBS washing, nuclear staining solution DAPI was added in the dark for 20 min incubation at 37°C at RT. The DAPI dye solution was discarded, and the wells were rinsed with PBS for observation under a fluorescence microscope (Zeiss CELL Observer SD).

### RNA Pull-Down

HD11 cells were spread in 6-well cell plates, and the cells were transfected with chLGP2, chLGP2 helicase, chLGP2 CTD, and chLGP2 K30A mutant (1 μg/well) at cell density 70–80%. Cells were infected with the IBV Beaudette strain after 24 h. Add protease inhibitors and RNase inhibitors to the cell lysate, and add the prepared lysate to the cells (200 μL/well). After 30 min of ice bath lysis, the cells in each well were collected in a 1.5 ml centrifuge tube. Centrifuge at 12000 rpm for 5 min at 4°C, take the supernatant, place it in a new 1.5 mL centrifuge tube, and place it in an ice-water bath. Pipette 20 μL of Anti-Flag gel suspension into the centrifuge tube with lysis buffer, incubate on ice, and shake for 2 h. Centrifuge at 6000 rpm for 30 s at 4°C, discard the supernatant, add 0.5 mL TBS and wash 3 times, place on a shaker in an ice bath for 5 min, repeat washing three times. Add 20 μL proteinase K to each tube, incubate at 42°C for 2 h, extract RNA.

### CO-IP and Mass Spectrometry

DF-1 and HD11 cells were spread in 6-well cell plates, and the cells were transfected with chLGP2 plasmid (1 μg/well) at cell density 70–80%. After 24 h, the cells were infected with the IBV Beaudette strain. The cell mixture was lysed and centrifuged at 6000 rpm for 3 min at 4°C. The obtained supernatant was transferred to a new centrifuge tube and added with 40 μL anti-Flag affinity gel magnetic beads. The mixture was incubated at 4°C overnight on a shaker. The next day, after careful washing with PBS 2–3 times, washed beads samples were collected and analyzed for the protein profile that interacted with chLGP2.

### Statistical Analysis

Data were analyzed with Graphpad Prism 9, and all values are expressed as mean ± standard deviation (SDM). Statistical analyses were conducted using Student’s *t*-test. P values < 0.05 were considered statistically significant.

## Conclusion

This article analyzes the role of chLGP2 in infected cells for the first time and found that chLGP2 can recognize and bind IBV RNA, and the structural integrity of chLGP2 and ATPase activity plays an important role in its inhibition. It is proved that chTRBP can combine with chLGP2 to inhibit the replication of IBV in cells. The molecular mechanism of chLGP2 inhibiting the proliferation of IBV is further analyzed. It is also the first study of the role of chTRBP protein in the process of IBV and avian virus infection.

## Data Availability Statement

The raw data supporting the conclusions of this article will be made available by the authors, without undue reservation.

## Author Contributions

KW and PC performed the experimental and computational work, coordinated the research, and contributed to the writing. Other authors have given some help and suggestions during the experiment and the writing of the manuscript. All authors contributed to the article and approved the submitted version.

## Conflict of Interest

The authors declare that the research was conducted in the absence of any commercial or financial relationships that could be construed as a potential conflict of interest.

## Publisher’s Note

All claims expressed in this article are solely those of the authors and do not necessarily represent those of their affiliated organizations, or those of the publisher, the editors and the reviewers. Any product that may be evaluated in this article, or claim that may be made by its manufacturer, is not guaranteed or endorsed by the publisher.
