# Supplementary material for: Chicken-Derived Pattern Recognition Receptor chLGP2 Inhibits the Replication and Proliferation of Infectious Bronchitis Virus
Source: Front Microbiol. 2022 Jan 25;12:810215. doi: 10.3389/fmicb.2021.810215 (PMC8824401; doi:10.3389/fmicb.2021.810215)
Supplement: Supplementary file 1 [file Table_1.DOCX]

SUPPLEMENTARY MATERIAL


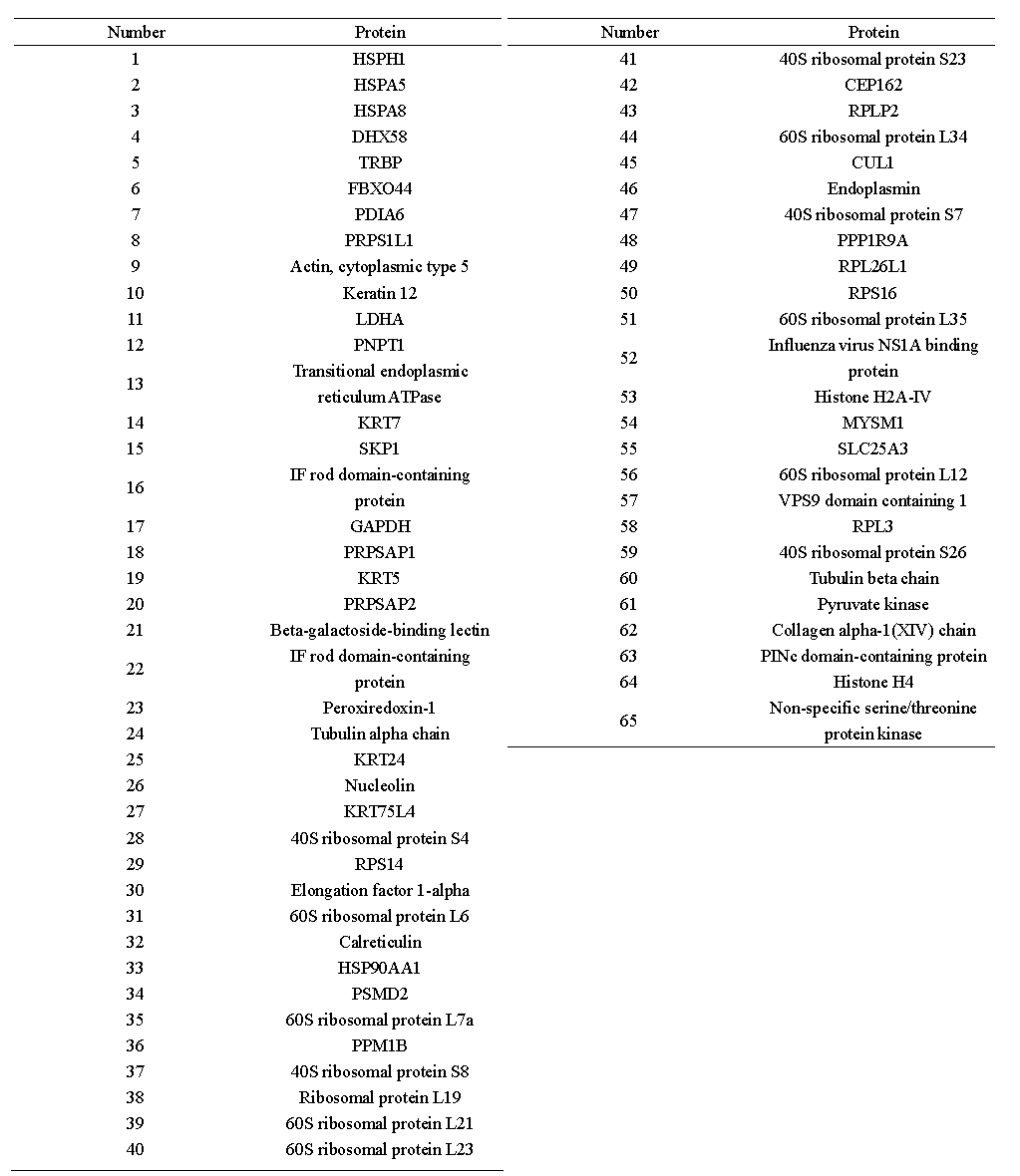


Figure S1: Total binding proteins of chLGP2.


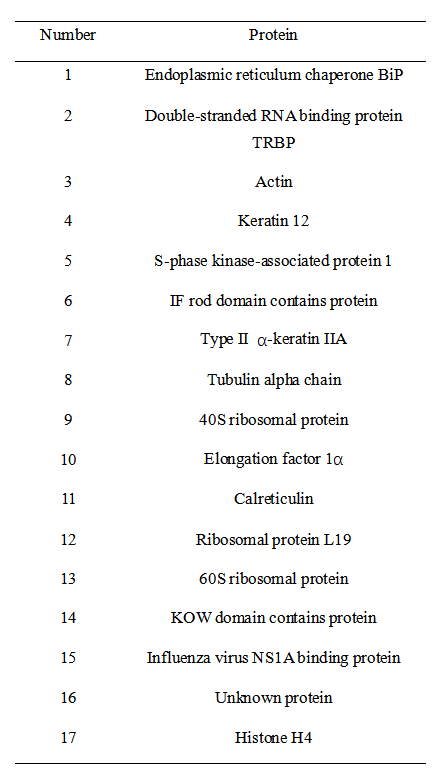


Figure S2: Seventeen proteins that bind to chLGP2 shared in HD11 and DF-1 cells.


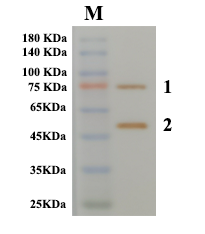


Figure S3: chLGP2 and chTRBP can interact with each other in HD11 cells. 1 represents chLGP2, 2 represents chTRBP.
